# Arbidol: The current demand, strategies, and antiviral mechanisms

**DOI:** 10.1002/iid3.984

**Published:** 2023-08-28

**Authors:** Yue Kang, Yin Shi, Silu Xu

**Affiliations:** ^1^ Jiangsu Key Laboratory of Neurodegeneration School of Pharmacy, Nanjing University of Chinese Medicine Nanjing Jiangsu China; ^2^ Department of Pharmacy Jiangsu Cancer Hospital & Jiangsu Institute of Cancer Research & The Affiliated Cancer Hospital of Nanjing Medical University Nanjing Jiangsu China

**Keywords:** antiviral strategy, arbidol, influenza virus, viral infection

## Abstract

**Background:**

High morbidity and mortality of influenza virus infection have made it become one of the most lethal diseases threatening public health; the lack of drugs with strong antiviral activity against virus strains exacerbates the problem.

**Methods:**

Two independent researchers searched relevant studies using Embase, PubMed, Web of Science, Google Scholar, and MEDLINE databases from its inception to December 2022.

**Results:**

Based on the different antiviral mechanisms, current antiviral strategies can be mainly classified into virus‐targeting approaches such as neuraminidase inhibitors, matrix protein 2 ion channel inhibitors, polymerase acidic protein inhibitors and other host‐targeting antivirals. However, highly viral gene mutation has underscored the necessity of novel antiviral drug development. Arbidol (ARB) is a Russian‐made indole‐derivative small molecule licensed in Russia and China for the prevention and treatment of influenza and other respiratory viral infections. ARB also has inhibitory effects on many other viruses such as severe acute respiratory syndrome coronavirus 2, Coxsackie virus, respiratory syncytial virus, Hantaan virus, herpes simplex virus, and hepatitis B and C viruses. ARB is a promising drug which can not only exert activity against virus at different steps of virus replication cycle, but also directly target on hosts before infection to prevent virus invasion.

**Conclusion:**

ARB is a broad‐spectrum antiviral drug that inhibits several viruses in vivo and in vitro, with high safety profile and low resistance; the antiviral mechanisms of ARB deserve to be further explored and more high‐quality clinical studies are required to establish the efficacy and safety of ARB.

## Graphical abstract

In this study, we review the structure of influenza virus, as well as the etiology, epidemiology, and pathophysiology of influenza virus. We describe the process of virus infection and summarize the current antiviral strategies and the reported antiviral mechanisms of ARB; these might be conducive to the discovery and development of novel antiviral drugs in the days to come.

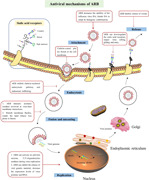

## 1 INFLUENZA VIRUS

### 1.1 Etiology and pathophysiology of influenza virus infection

Influenza virus infections have become a major threat to public health and are accountable for high morbidity and mortality annually.^1^ They can be mainly transferred through air droplets, contact with susceptible and infected individuals, or contact with contaminated objects. The typical clinical symptoms of influenza virus infections are a sudden outset of acute high fever, aching muscles, fatigue, and numerous other respiratory symptoms.^1^

**Figure 1:**
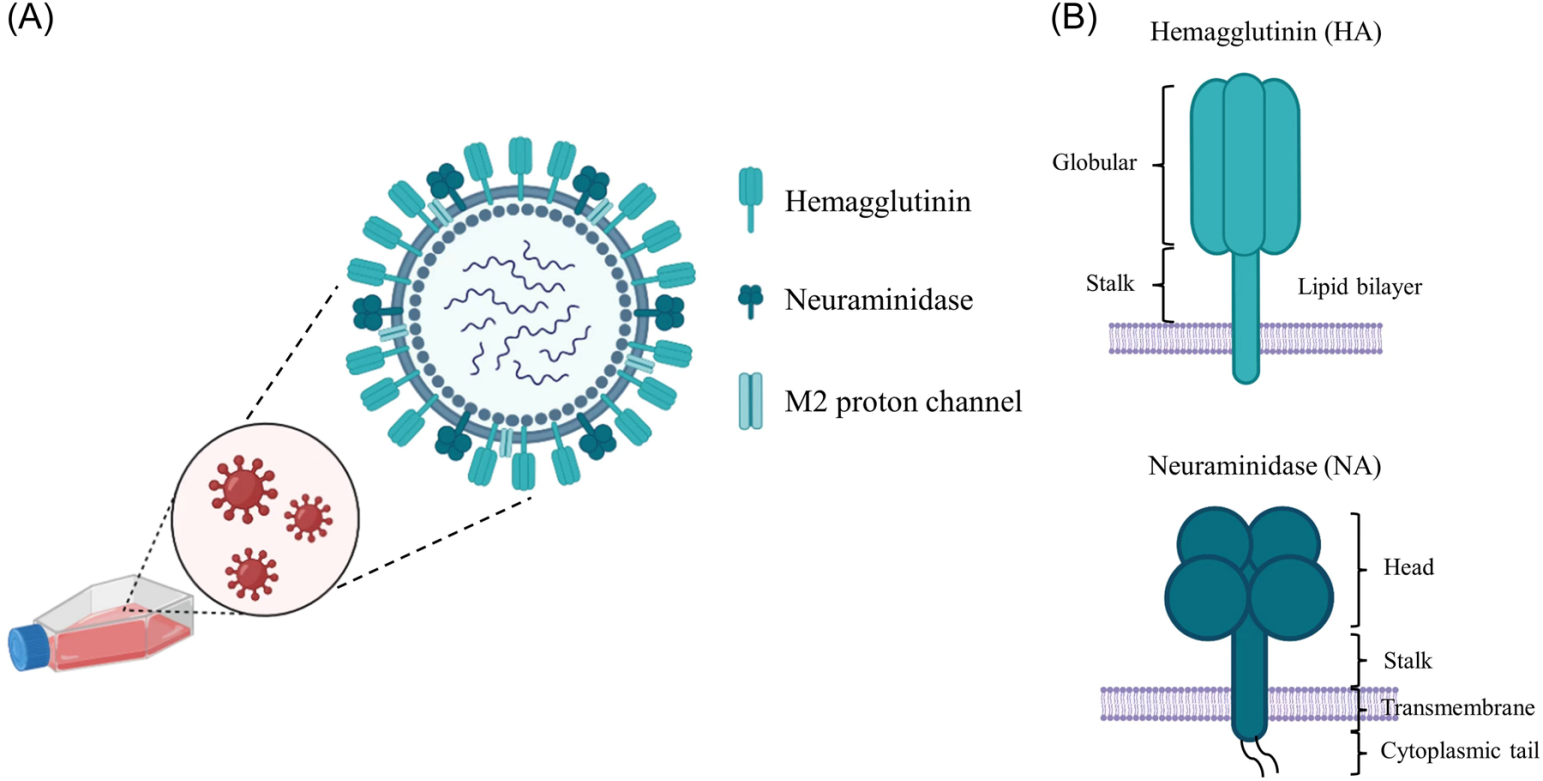
Graphic representation of influenza virus. (A) The structure of influenza virus. (B) Influenza virus hemagglutinin (HA) is structurally like a trimer composed of the globular and stalk domain. Neuraminidase (NA) is a tetramer consisting of four monomers, including head, stalk, transmembrane, and cytoplasmic tail.

Influenza viruses are eight‐segmented, enveloped RNA viruses with a diameter of 80–120 nm and comprise three parts: envelope, matrix protein (MP, including M1 and M2 protein), and core^2^, ^3^, ^4^ (Figure 1A). The outer surface of the influenza virus contains two types of glycoprotein protrusions, hemagglutinin (HA) and neuraminidase (NA),^5^, ^6^, ^7^ which are the main components of the antigenic structure of the influenza virus. HA, comprising HA1 and HA2 units, is a stable trimer with a highly glycosylated head and stalk (Figure 1B). HA assists the virus to adsorb on the host cell membrane by attaching to the sialylated glycan receptors and further triggers the virus uptake.^5^ NA is a tetramer comprising four polypeptides and is the main enzyme in the process of entry, replication, maturation, and release of influenza virus. The NA head is active and can be detached from the virus via proteolysis.^8^, ^9^, ^10^ Based on the antigenicity of HA and NA, the same class of virus can be classified into multiple subtype strains. HA antigens have 18 subtypes (HA1–HA18), whereas NA antigens have 11 subtypes (NA1–NA11).^11^ The main subtypes prevalent among human beings are HA1–HA3, and NA1 and NA2.^5^, ^12^, ^13^ The antigenic structure of the influenza virus makes it prone to recombine and mutate and produce novel subtypes during the virus replication process due to the segmented genomes.^14^

### 1.2 Influenza virus invasion

**Figure 2:**
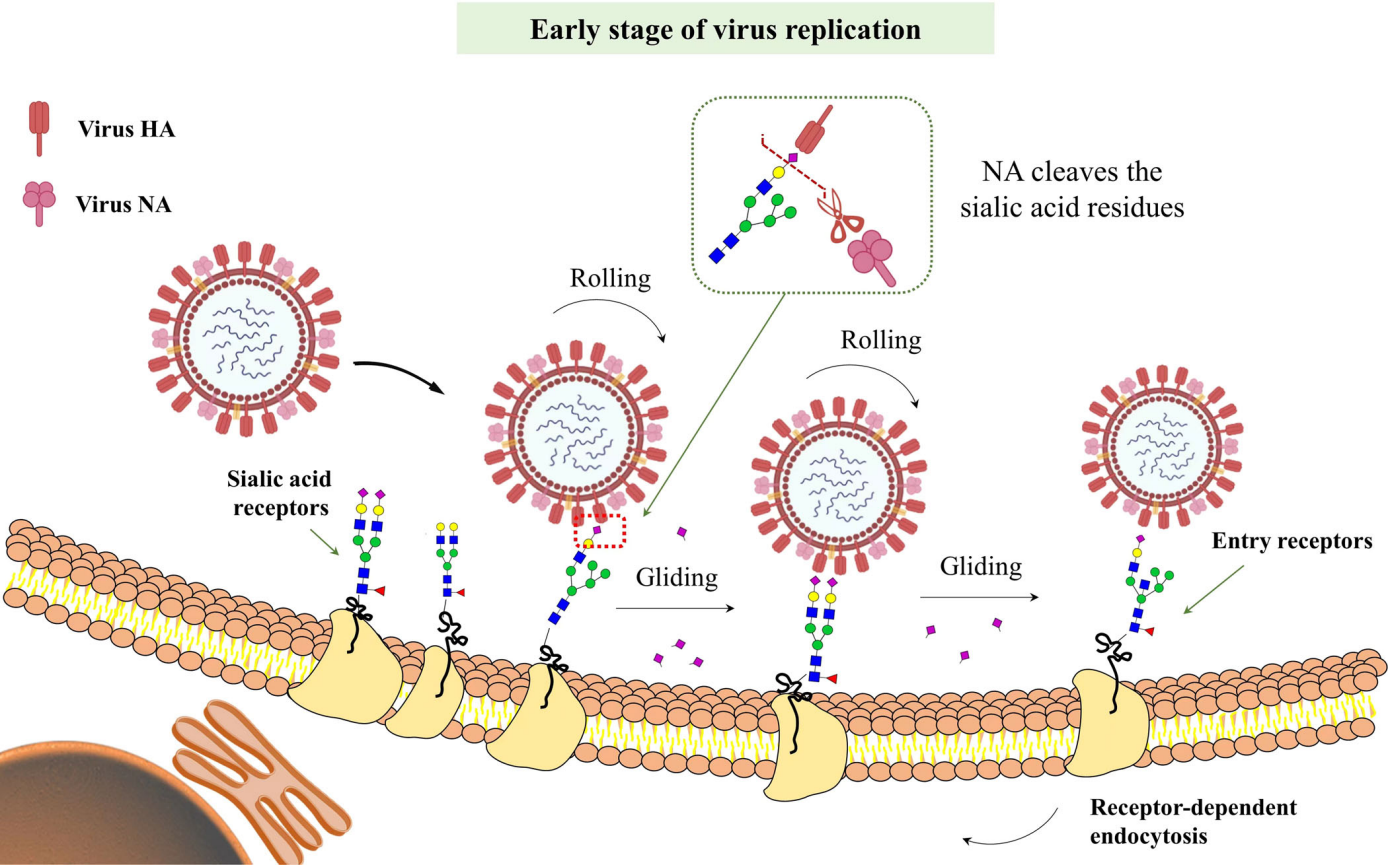
Schematic representation of early stages of virus replication. During the early stages of virus replication (0–2 h), when the virus attaches the host cell membrane, the virus continuously rolls and glides on the cell surface via a hemagglutinin (HA)‐receptor exchange mechanism, virus HAs bind to sialic acid (SA) receptors, and neuraminidase (NA) protein cleaves SAs, leading to the density of SA receptors around the binding sites to decrease. The density gradient of SAs would promote virus rolling and gliding until the virus finds an appropriate entry receptor, followed by successful invasion by endocytosis pathway.

During the early stages of virus replication (0–2 h), virus movement plays a significant role in the virus invasion, depending on the cooperation of HA affinity and NA activity.^10^, ^15^ The human influenza virus has a high affinity to *α*2,6‐linked sialic acids (SAs) receptors, whereas the avian influenza virus attaches to *α*2,3‐linked SA receptors in the hosts. However, sometimes, the attachment of HA to SA receptors does not lead to a successful invasion. NA repeatedly cleaves the SAs by catalyzing the cleavage of *α*2,6 or *α*2,3 linkage between the terminal SA residues and adjacent oligo lactose, leading to loose HA‐receptor interactions, encouraging virus crawling and gliding until it meets its appropriate entry receptors, and finally triggering the virus uptake and entry receptors into hosts (Figure 2). During the early stages of infection, the receptor exchange mechanism acts as the driving force to confirm virus migration on the cell surface; a stable balance between the HA and NA activities is favorable for successful infection.^16^, ^17^

After HA encounters appropriate entry receptors, the virus particles are taken into hosts. Viruses invade organisms via the clathrin‐mediated endocytosis pathway, during which the cell membrane forms invaginated phagocytic vesicles known as clathrin‐coated pits; the virus particles can penetrate the cytoplasm via the clathrin‐mediated endocytosis pathway.^18^, ^19^, ^20^ The downstream signaling required for internalization is subsequently activated. Under an environment of low pH (pH 5), the virus envelope fuses with the cell membrane via the HA molecular rearrangement; the exposed fusion peptide is inserted into the targeted membrane, making the viral and endosomsal membranes move closer. M1 protein connects the viral membrane with the viral ribonucleoproteins (vRNPs) in the core, which defines the virus shape. M2 proteins are responsible for transferring viral nucleocapsids to the correct locations.^3^, ^21^, ^22^ The virus sheds the shell in the cytoplasm, during which vRNPs are released from the capsid. HA fuses viral endosomal membranes via the cleavage of HA by host cell proteases into two subunits, HA1 and HA2. This cleavage is needed to facilitate the exposure of the fusion peptide on the N‐terminus of HA2 on pH change in the endosome. The exposed fusion peptide inserts in the endosomal membrane, whereas the C‐terminal transmembrane structural domain anchors HA2 to the virus membrane, forming a hairpin preconfiguration. Next, HA2 forms a hairpin by folding itself, making two membranes closer. The hairpin bundle further folds into a six‐helix bundle and the two membranes are closely bound together, leading to the formation of a lipid stalk and subsequent fusion of the two membranes. The subsequent genetic instructions, nucleic acids, and proteins are synthesized.^23^, ^24^ Using viral nucleic acid as the template, novel nucleic acid molecules are synthesized under the action of enzymes; the transcription product of viral RNA or DNA, messenger RNA, synthesizes capsid protein on ribosomes.

In the late stages of infection (6–10 h), most of the progeny viruses mature via endoplasmic reticulum processing. Viruses are transported through the mucus and destroy the SA receptors on the host cells, encouraging the release of progeny virus particles from the infected hosts. However, mature influenza virus particles still depend on SA residues for connecting with hosts through *α*2,6 or 2,3 glycosidic bonds, preventing the immediate detachment of the influenza virus. NA is responsible for catalyzing the hydrolysis of these important glycosidic bonds; mature virus particles finally detach from the host cells to infect new epithelial cells, spreading influenza viruses in the host.^9^, ^19^, ^25^

## 2 CURRENT ANTIVIRAL STRATEGIES

**Figure 3:**
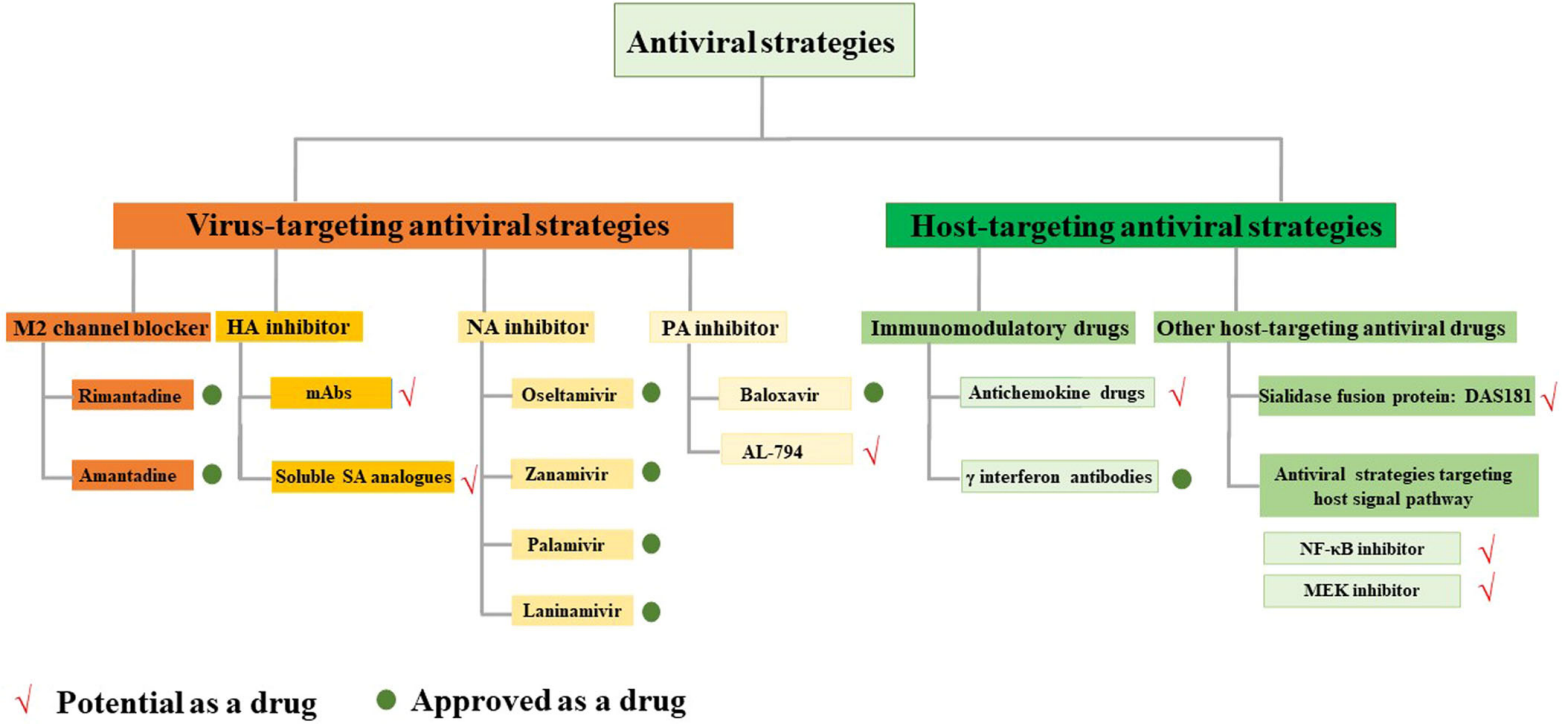
The current antiviral strategies. HA, hemagglutinin; mAbs, monoclonal antibodies; NA, neuraminidase; NF‐κB, nuclear factor‐κB; PA, polymerase acidic protein; SA, sialic acid.

Current antiviral methods are mainly focused on two strategies: directly targeting the virus itself or its hosts. Antiviral drugs targeting viruses mainly affect some stages of the viral replication cycle for protecting against virus invasion, such as interrupting virus adsorption, blocking membrane fusion, inhibiting polymerase and protease, or impairing viral release (Figure 3), whereas host‐directed antivirals can treat influenza by regulating immunity, interfering with and inhibiting viral replication by drug targets acting on host cells.^26^ According to antiviral mechanisms, the drugs can be classified into HA inhibitors, M2 channel blockers, NA inhibitors, and polymerase acidic protein (PA) inhibitors.^2^

### 2.1 HA inhibitors

The most important step of influenza A virus infection is the attachment of HA to SA receptors on the cell surface; thus, HA inhibition is an attractive and promising antiviral strategy.^27^, ^28^ Monoclonal antibodies (mAbs) that can target the globular head of HA protein can act as inhibitors for influenza viruses. Treatment with mAbs effectively inhibits virus replication both in vitro and in vivo.^29^ mAbs have shown the desired effect against various strains of influenza virus after structural modifications. To date, five mAbs (CR6261, CR8020, VIS410, MEDI8852, and CT‐P27) have shown promising efficacy and safety in Phase II clinical trials.^30^, ^31^, ^32^, ^33^, ^34^ Moreover, many soluble SA analogs and peptide mimics are recommended as potential influenza virus inhibitors: soluble SA analogs can block the receptor binding pocket of virus HA, whereas SA peptide mimics interact with the receptor binding pocket to inhibit virus entry. Seasonal H1N1 has been reported to be blocked using soluble SA analogs,^35^ indicating they hold good development prospects.

### 2.2 M2 channel blockers

M2 channel blockers, including amantadine and rimantadine, are common antiviral drugs that can inhibit the M2 ion channel activity for preventing the replication of the influenza virus.^36^ Most influenza A viruses carry S31N mutation in their *M2* genes^37^; UAWJ280, a novel deuterium‐containing M2‐S31N inhibitor, demonstrated antiviral effect in mice infected with oseltamivir (OSV)‐sensitive or drug‐resistant influenza A viruses.^38^ In 2021, the efficacy of two adamantane azaheterocyclic rimantadine derivatives on pneumonia caused by the rimantadine‐resistant influenza A virus in mice was reported.^39^ However, M2 channel inhibitors are only effective for the initial infection or aggregation of influenza A and not influenza B. More and more adverse reactions of the central nervous system or gastrointestinal tract have recently been reported during treatment. Since the seasonal influenza in 2005 in the United States, M2 channel blockers are not being prescribed for the treatment or chemical prevention of the influenza virus due to the rapid mutation of the viral genome.

### 2.3 NA inhibitors

NA inhibitors are a type of antiviral drugs that can block the activity of viral NA protein.^25^ The function of NA inhibitors is to prevent the release of viruses from the infected hosts, or to impede the replication of the virus in the organism. Unlike M2 channel blockers, NA inhibitors are effective for both influenza A and B viruses. So far, marketed NA inhibitors include OSV, zanamivir, laninamivir, and peramivir. Except for laninamivir licensed only in Japan, the other three are licensed worldwide for the treatment and prevention of influenza infections.^40^ They can relieve the symptoms of influenza virus infection, shorten the disease course, and reduce complications and toxicity. OSV has been approved for clinical application in the United States since 1999 and is considered to be the first oral NA inhibitor available.^41^, ^42^ OSV acts by blocking the active sites of NA, preventing the release of viruses from the hosts. The common adverse drug reactions associated with OSV are allergic reactions, arrhythmia, and vomiting. Nevertheless, OSV is the only drug approved as a stock drug for an influenza pandemic. Zanamivir is the first commercially developed drug clinically used for the treatment of both influenza A and B viruses. It is a competitive inhibitor of viral NA and prevents the sialidase activity of NA. Zanamivir is a prescription inhalation antiviral drug approved in the US since 1999 and approved for influenza virus prevention in 2006. The most frequent side effects of zanamivir are headaches, nausea, and cough.^43^, ^44^ Although NA inhibitors have been widely used clinically for decades, the efficacy of these drugs has been endangered by the emergence of drug resistance due to the highly mutated virus genome. In 2004, high rates of OSV‐resistant influenza virus infection were reported. From 2008 to 2009, seasonal H1N1 cases with high resistance to OSV were seen all over the world. Moreover, genes expressing resistance to zanamivir were tested in critically ill patients infected with H7N9. It has been shown that the H274Y mutation reduces the binding potency of OSV to NA by inhibiting conformational changes in Glu276.^45^ In recent years, some novel NA inhibitors (AV5080, CS‐8958, and HNC042) are conducting clinical trials to evaluate the effectiveness and safety of influenza treatment, and might be promising to overcome the challenge of OSV‐resistant virus strains.^26^

### 2.4 PA inhibitors

The RNA polymerase complex essential for virus replication and transcription comprises three units: polymerase basic protein 2, polymerase basic protein 1, and PA. Specific residues of PA are highly conversed and mutated, making endonuclease inactive. The antiviral agent baloxavir marboxil (hereafter baloxavir) was developed to specifically target PA for inhibiting its activity but not affect polymerase basic protein 1 and 2 activities. Baloxavir has demonstrated broad‐spectrum antiviral effects against many subtypes of influenza A viruses including strains with NA inhibitor‐resistant mutations. Baloxavir has currently been under phase III clinical trials and has shown the desired effect; oral administration of baloxavir had minor side effects and was effective in relieving influenza virus symptoms with a halftime of around 80 h. It was approved for specific treatment of uncomplicated influenza in patients in the United States and Japan in 2018.^46^ Several investigations have reported reduced susceptibility to baloxavir for a variety of influenza viruses in the United States and the Asia‐Pacific region.^47^, ^48^, ^49^ AL‐794 is an orally active isobutyrate prodrug of ALS‐033719 that selectively binds to the PA endonuclease structural domain and effectively inhibits endonuclease activity. In a phase I clinical study, the results showed that AL‐794 had dose‐dependent antiviral activity with no resistance was observed.^50^

### 2.5 Host‐targeting antivirals

Considering the emergence of drug‐resistant viruses, it is still important to look for novel targets targeting host cellular factors.^51^ Immunomodulatory drugs can suppress the severity of influenza diseases by protecting the body from excessive inflammatory reactions. Cyclooxygenase‐2 (COX‐2), a component of arachidonic acid cascade, is considered to participate in the process of inflammation and immune response. Therefore, it can serve as the target of antiviral strategy. A phase III clinical trial of celecoxib, a classic COX‐2 inhibitor, compared with OSV for the treatment of severe influenza A is underway. Other compounds that inhibit the expression of chemokines and cytokines can also reduce the inflammatory response to infection, such as antichemokine drugs and gamma interferon antibodies.^52^, ^53^, ^54^ In addition, some drugs targeting on host cells can also interfere with and inhibit virus replication. DAS181, a novel sialidase fusion protein, can block the entry process of influenza virus by destroying the SA structure on the surface of host cell membrane, and its efficacy and safety have been verified in clinical trials.^55^ The broad‐spectrum antiviral inhibitor iminosugars can be used as ERα‐ competitive inhibitors of glucosidase I and II, which are host cell enzymes that can remove glucose residues from high mannose N‐linked glycans attached to glycoproteins, thus facilitating proper protein folding and transport within the cell. Iminosugar UV‐4 has been proven to be an effective antiviral drug against dengue fever^56^ and influenza virus in vivo.^57^ Due to the considerable complexity of the host cell‐dependent viral replication process and the body's response process, other new targets (nuclear factor‐κB signal pathway, Raf/MEK/ERK signal pathway) and drugs have been discovered in recent years.^58^, ^59^, ^60^

## 3 ARBIDOL (ARB)

**Figure 4:**
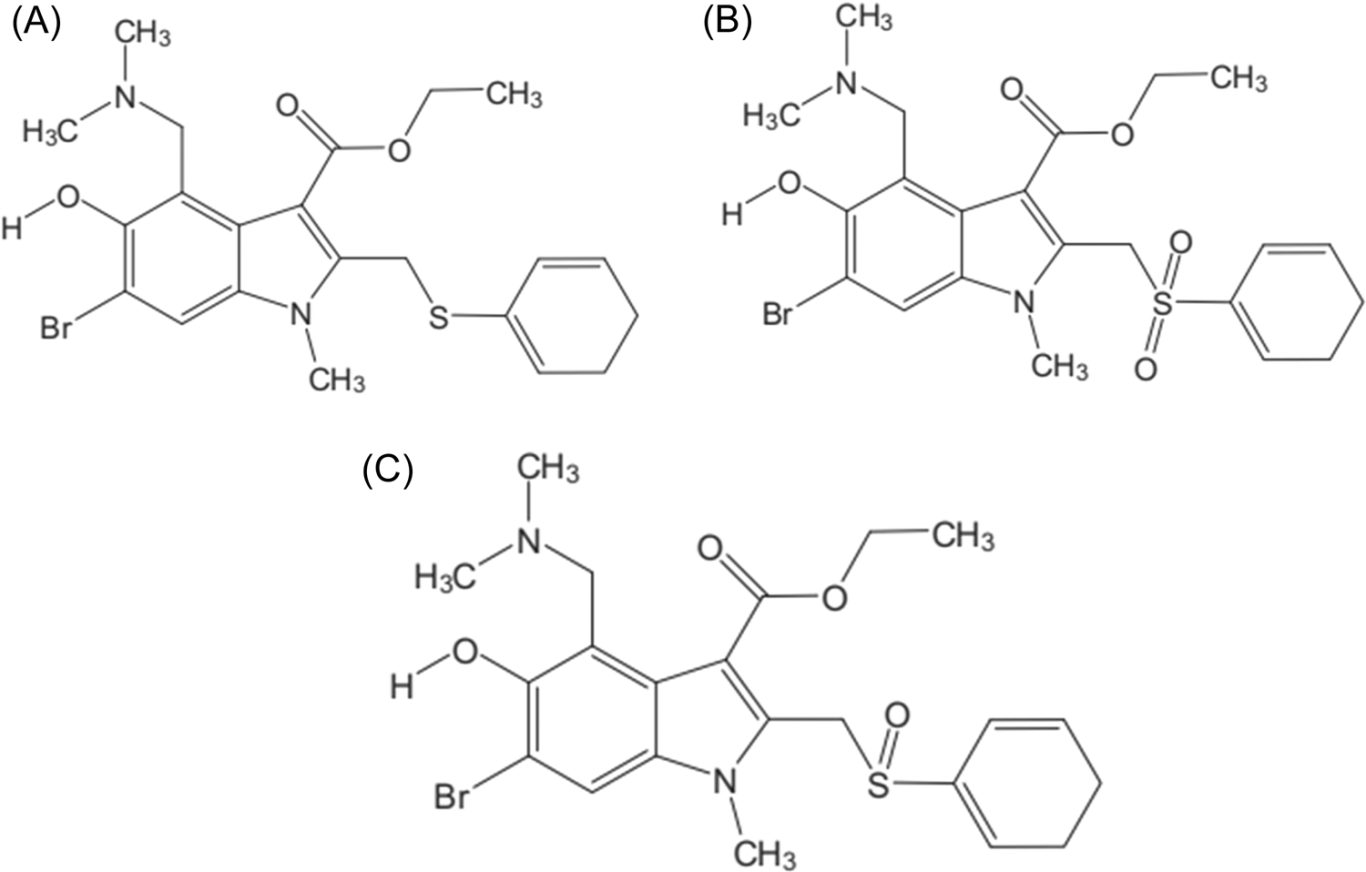
The structures of arbidol (A), sulfonyl‐arbidol (B), and sulfinyl‐arbidol (C).

ARB is a small‐molecule indole‐derivative developed in Russia^61^ and marketed in Russia and China in 1993 and 2006, respectively, with approved indications for the prophylaxis and treatment of influenza A and B viruses. ARB has a carboxylate structure and therefore it can be utilized as a carrier precursor drug (Figure 4), the drug possessing this structure is mostly lipophilic, which can be used as a hydrolytic substrate to form active compounds and accumulate in the body. ARB is a broad‐spectrum antiviral drug that inhibits several viruses in vivo and in vitro,^62^, ^63^ including influenza A, B, and C viruses, severe acute respiratory syndrome coronavirus (SARS‐CoV), Middle East respiratory syndrome coronavirus, SARS‐CoV‐2, Coxsackie virus (CV), respiratory syncytial virus (RSV), Hantaan virus, herpes simplex virus (HSV), and hepatitis B and C viruses.^64^, ^65^, ^66^ ARB is a clinically proven antiviral drug with good clinical application prospects, owing to its low side effects, high safety profile, and low resistance.

### 3.1 Antiviral mechanism of ARB

#### 3.1.1 ARB inhibits virus entry and membrane fusion

**Figure 5:**
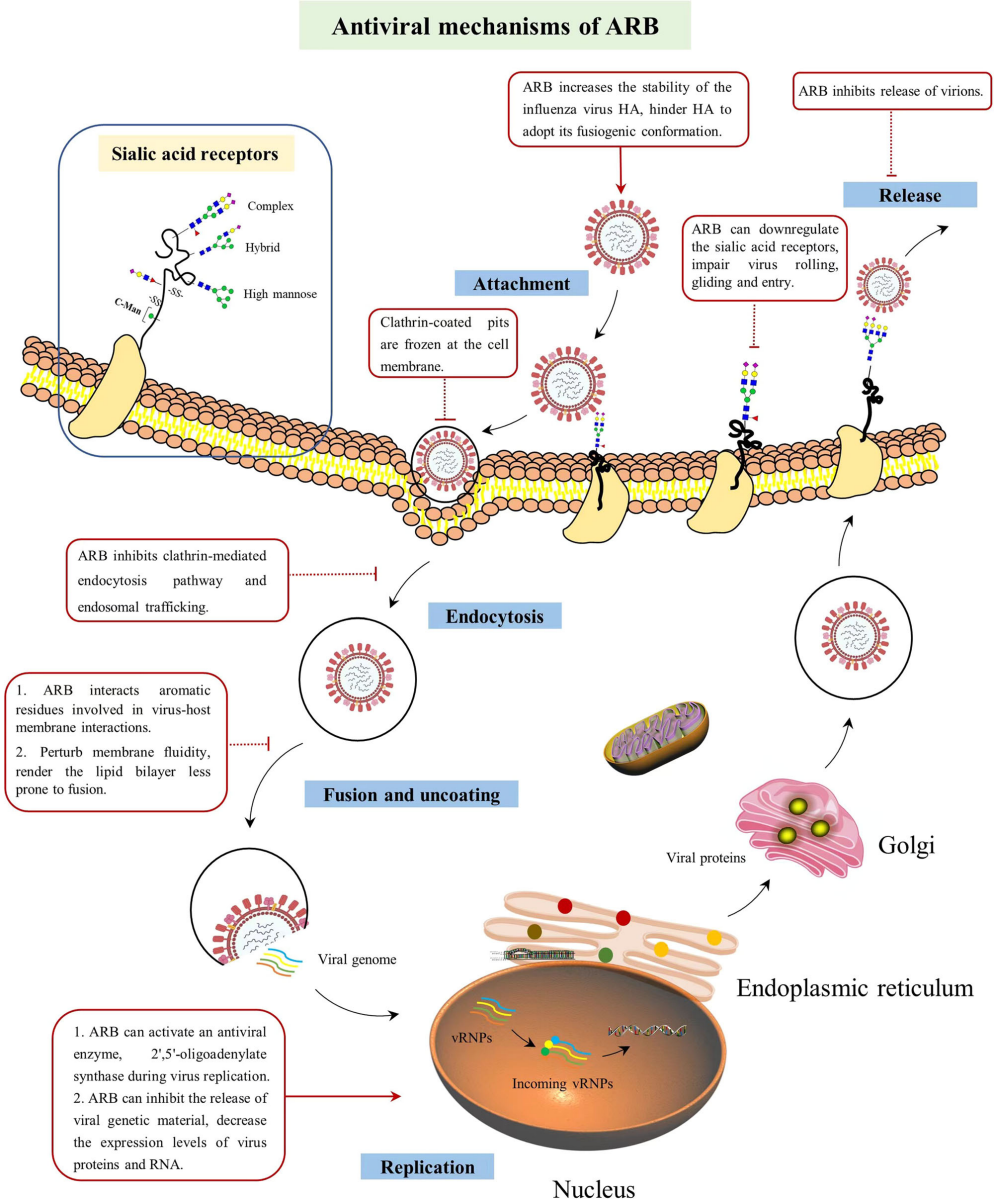
The molecular mechanisms of arbidol (ARB). ARB inhibiting different steps of virus replication cycle were indicated in the red boxes.

The broad‐spectrum antiviral activity of ARB specifies that it acts on the important steps of virus–cell attachment. Moreover, ARB plays a direct role in removing the influenza virus, which is similar to the role of other direct‐acting antiviral drugs. The inhibitory effect of ARB on different stages of the virus life cycle, such as cell entry, attachment, internalization, and replication, has also been reported (Figure 5). Therefore, ARB can also be used as a host‐targeting agent.^64^, ^65^ ARB can interfere with virus entry or the membrane fusion of virus and host to block virus invasion. When used before the infection, ARB exerts maximal antiviral activity, which highlights its activity in the early steps of viral infection and/or the requirement for ARB to impregnate cells. ARB interferes with influenza virus entry through at least one of the following factors: Rab5, dynamin‐2, actin, or acidification.

**Figure 6:**
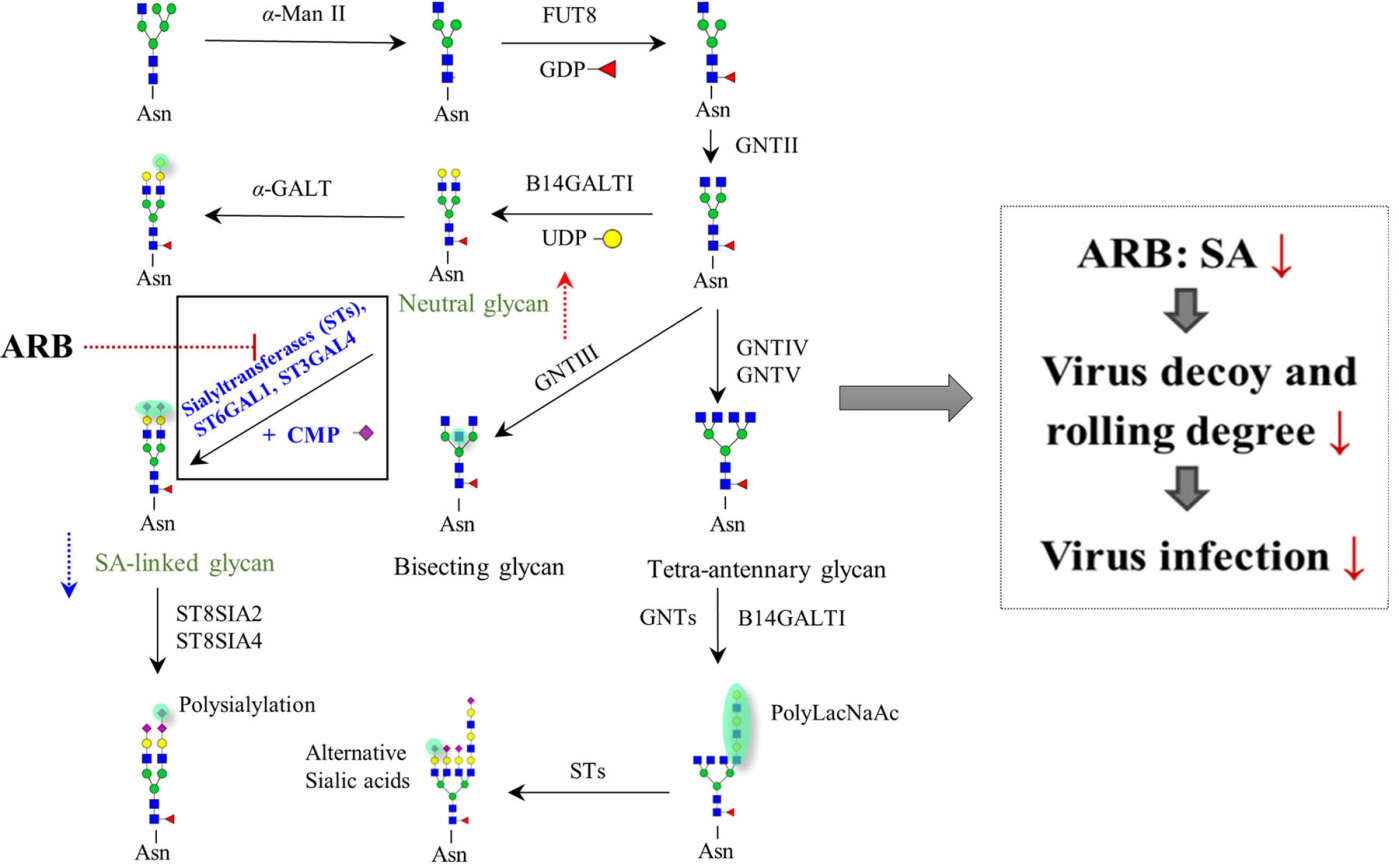
Schematic representation of the involvement of arbidol (ARB) inhibition within the mammalian *N‐*glycosylation processing pathways. Treatment of ARB can inhibit the expressions of sialyltransferase (ST), leading to the downregulation of sialic acid (SA)‐linked *N‐*glycans on the cell membrane, making the virus utilization on SA‐linked *N‐*glycans impaired, and resulting in the receptor‐dependent rolling and entry capacity of virus significantly retarded, thereby inhibiting virus rolling, gilding, and entry.

During the early stages of infection, after attaching to HA receptors on the cell membrane, the influenza virus is internalized into clathrin‐coated vesicles and taken into the host cell via clathrin*‐*mediated endocytosis. Virus binding to the plasma membrane is severely inhibited following ARB treatment. Subsequently, ARB can also directly impair clathrin*‐*mediated endocytosis by preventing the release of vesicles or interrupting dynamin‐2‐induced membrane rupture, leading to the accumulation of clathrin‐coated vesicles or failure of viral transportation in cells, inhibiting virus infection at the entry step.^67^

Recently, a novel antiviral mechanism of ARB was revealed in our study (Figure 6).^68^ Using the advanced TiO_2_‐PGC chip quadrupole time‐of‐flight mass spectrometry method, we found that the SA‐linked *N‐*glycans levels that act as SA receptors significantly decreased in PR8 virus infection. In contrast, the neutral *N‐*glycans levels increase due to the cleavage of SA‐linked *N‐*glycans by virus NA protein when the virus rolls and glides on the cell surface at the early step of infection; after that, the virus is successfully uptaken into cells when it meets an appropriate entry receptor. Pretreatment with ARB can remarkably downregulate SA‐linked *N‐*glycans on the cell surface by obstructing the expressions of sialyltransferases, which is related to the biosynthesis of SA‐linked *N‐*glycans. This downregulation of SA‐linked *N‐*glycans by ARB significantly reduces the virus utilization on SA‐linked *N‐*glycans, and receptor‐dependent rolling and entry capacity of the virus are significantly decreased, thus inhibiting virus rolling, gilding, and entry.^68^

Mechanistically, ARB is a hydrophobic molecule with an indole structure, which is prone to forming supramolecular arrangements by interacting with the aromatic amino acid residues of viral glycoproteins, leading to the interference of membrane fusion. Moreover, ARB can interact with important residues in viral lipid membranes, which may underlie the antiviral effects of ARB.^65^, ^69^, ^70^, ^71^ ARB can also interact with phospholipids on the cell membrane,^72^ which may affect the fluidity of the membrane, inhibiting lipid bilayer fusion,^65^, ^73^ hindering the delivery of virus particles to correct locations, and blocking the process of membrane fusion. All these findings underlie the direct‐acting antiviral and host‐targeting effects of ARB.

An acidic environment (pH 5.0) is needed for the membrane fusion of the influenza virus and host cells, and changes in pH will hinder the fusion process. ARB is a weak alkaline drug that can increase the pH of the intracellular environment, making the virus unable to complete the process of membrane fusion and release the virus genome, resulting in effective inhibition of virus infection.^74^ Moreover, ARB directly interacts with all virus HA subtypes, among which H7 has the strongest affinity; ARB dwells in a discrete binding capsule,^75^ forming an ARB‐HA complex and enhancing the structural stability of HA, inhibiting the pH‐induced transition of HA into its functional fusogenic state, resulting in failure of membrane fusion.^64^, ^67^, ^76^, ^77^

#### 3.1.2 ARB inhibits virus replication

ARB can inhibit influenza virus replication; enveloped RNA viruses are more sensitive to ARB than nonenveloped DNA viruses.^78^ The expression levels of virus proteins and RNA in ARB‐treated cells gradually decreased; the cells can be treated by replicating virus RNA after weeks of ARB treatment. During viral replication, the enzyme activity of 2′,5′‐oligoadenylate synthase, an antiviral enzyme, is inhibited, and ARB changes the permeability of the cell membrane when it enters the nucleus and activates 2′,5′‐oligoadenylate synthase, which interferes with cell metabolism and prevents virus replication.^78^, ^79^ Under physiological conditions in hosts, ARB can inhibit the fusion of the viral lipid cytoplasmic sheath with the cell membrane and the release of viral genetic material. Moreover, ARB can significantly activate macrophage phagocytosis, which can enhance the resistance of the organism to viruses. During the process of replication, an internal membrane environment favorable for virus replication is created, and ARB can prevent the formation and maintenance of a membrane network, resulting in the prevention of virus replication.^73^ Furthermore, lipid bilayers are important factors for virus replication. The replication complex distributed on the cell membrane is known as vacuoles; ARB can impair these vacuoles to terminate the replication process.^64^, ^65^

#### 3.1.3 Immunomodulatory activities of ARB

ARB has immunomodulatory, interference‐inducing, and antioxidant properties.^62^ It can stimulate host cells to produce interferons, stimulate humoral responses, and activate macrophages and cellular and humoral immunity.^80^, ^81^ After ARB treatment, the serum interleukin (IL)‐1β, IL‐6, IL‐12, and tumor necrosis factor α (TNF‐α) levels significantly decreased in the bronchoalveolar lavage fluids and lung tissues of mice.^69^ ARB can decrease the pathological changes in the infected tissues and modulate the serum TNF‐α levels to fight against viruses.^82^, ^83^ Semenenko et al.^84^ found that in the absence of HLA‐DR positive expression and major immunoglobulin, ARB can normalize several immune regulatory indicators and the phagocytic functions of cells and upregulate the expression of CD3, CD4, and CD16, leading to the activation of immune activity. In the study of the immunomodulatory effect of ARB on mice, ARB induced the production of interferons in vivo and enhanced nonspecific, humoral, and cellular immune function in normal and immunocompromised mice.

### 3.2 Efficacy of ARB

#### 3.2.1 Anti‐influenza virus

ARB effectively inhibits influenza A (H1N1, H2N2, H3N2, H5N1, and H9N2),^64^, ^85^, ^86^ B, and C viruses^87^ in vivo or in vitro. In vitro studies have reported that the half maximal inhibitory concentration (IC50) of ARB is 2.5–16 μg mL^−1^, and that ARB has a comparable or stronger inhibitory effect on virus replication than other anti‐influenza drugs, including amantadine, ribavirin, and OSV.^64^, ^88^, ^89^, ^90^ ARB has a strong inhibitory effect on amantadine‐sensitive and ‐resistant strains and OSV‐resistant strains of viruses.^91^ ARB in combination with amantadine can enhance the inhibitory effect of influenza viruses.^84^, ^86^

ARB has a good prophylactic effect against influenza virus infection. In a Russian randomized controlled trial,^92^ ARB 200 mg d^−1^ was orally administered for 10–18 days to a group of workers during an influenza A epidemic and was found to display a significant preventive effect. A Russian study confirmed that prophylactic oral ARB administered to military personnel reduced acute respiratory viral infections and the incidence of mixed bacterial and viral pneumonia.^93^ ARB demonstrates a preventive effect on influenza and other acute viral respiratory infections in patients with underlying respiratory diseases. In a recent Russian study, oral ARB administered to patients with asthma and chronic obstructive pulmonary disease (COPD) for preventing viral infections reduced the incidence of influenza and other acute viral respiratory infections in patients with asthma and COPD, the frequency and severity of exacerbations in patients with COPD, and the number of hospitalizations.^94^

On the other hand, oral administration of ARB early during the onset of influenza shortens the duration of illness and also reduces symptoms. A study by Kiselev et al.^95^ showed that all symptoms were resolved within the first 60 h after therapy initiation in 23.8% patients with laboratory‐confirmed influenza in the ARB group and it was 5.7 times greater compared with placebo group (4.2%) (*p* < .05). Severity of illness, catarrhal symptoms, and intoxication was reduced with ARB compared with placebo. A 2019 study on the safety and efficacy of ARB treatment, including 359 patients with influenza or acute upper respiratory tract infection randomized into treatment group (ARB) and placebo group, showed that influenza symptoms were milder and of shorter duration in the treatment group (including duration of fever [*p* = .023], duration of muscle pain [*p* = .037], and duration of weakness [*p* = .008]). Furthermore, the incidence of adverse events was similar in both groups.^96^

ARBs are effective in preventing and treating influenza in children. A study found that in children, ARB prevented the onset of influenza and other respiratory viral infections, reduced the severity of illness, and decreased the occurrence of other complications.^97^ In a Russian randomized double‐blind study of ARB treatment in children aged 1–14 years with influenza and other respiratory viral infections, ARB reduced the incidence and duration of the symptoms of catarrh and intoxication; no complications were noted in the ARB‐treated group, whereas in the control group, two children had septic otitis media and one child had pneumonia.^98^

#### 3.2.2 Anticoronavirus

SARS‐CoV‐2 is a positive‐sense single‐stranded RNA capsid virus with a high degree of gene sequence homology and belongs to the same genus of betacoronavirus as the SARS‐CoV outbreak in 2003. The main pathogenic mechanism of SARS‐CoV‐2 is to invade host cells by binding to angiotensin‐converting enzyme 2 receptors on the surface of spike glycoprotein,^99^ resulting in tissue or organ damage and activating the body's immune system to induce an extreme immune inflammatory response, forming a “cytokine storm” and leading to multiorgan damage.

ARB inhibits the fusion of SARS‐CoV‐2 with the cell membrane by dual binding to the receptor‐binding domain and angiotensin‐converting enzyme 2 receptors of SARS‐CoV‐2 spike protein on the host cell membrane; moreover, it interferes with the acidification of endocytic vesicles in the endocytic pathway and inhibits the intracellular release of viral particles. The immunomodulatory effect of ARB may inhibit the excessive release of inflammatory factors, such as IL‐6, in the lung tissues and reduce the chances of inflammatory lung injury.^62^

In vitro studies have shown that ARB has very favorable antiviral effects against SARS‐CoV‐2, showing statistically significant decreases in the binding efficiency and inhibition of viral entry and postviral events.^100^ However, the results of clinical trials have been inconclusive.

ARB alone and in combination with other drugs have good efficacy in reducing the viral load of patients after admission and shortening the time for SARS CoV‐2 nucleic acid to turn negative. In a retrospective study conducted in China comparing 36 patients treated with lopinavir/ritonavir with 16 patients treated with ARB, ARB monotherapy was more effective than lopinavir/ritonavir, as all patients treated with ARB developed undetectable viral load by day 14 postadmission compared with 55.9% of patients treated with lopinavir/ritonavir (*p* < .01). Moreover, the duration of positive RNA testing was shorter in the ARB group than in the lopinavir/ritonavir group (*p* < .01).^101^ For combination therapy, a randomized controlled trial in Iran recruited 100 patients: 50 were treated with dual therapy with ARB +hydroxychloroquine and 50 were treated with lopinavir/ritonavir and hydroxychloroquine.^102^ This study found that ARB significantly contributed to laboratory and clinical improvements, including higher peripheral oxygen saturation on the seventh day of treatment (*p* = .02), less severe involvement on chest computed tomography scan after 30 days (*p* = .004), and shortened the length of stay (*p* = .02). However, other parameters such as the need for intubation (*p* = .6), the need for mechanical ventilation (*p* = .6), and the time to defervescence or mortality (*p* = .5) did not show significant differences. Deng et al.^103^ compared a cohort of patients treated with a combination of ARB and lopinavir/ritonavir with a control group that received only lopinavir/ritonavir in their retrospective study. They observed a higher rate of viral negativity in the combination therapy group on days 7 and 14, and significant improvement in chest computed tomography on days 7 (*p* < .05).^103^ In another retrospective cohort study, ARB in combination with interferon α2b (IFN‐α2b) was compared with IFN‐α2b monotherapy, and the combination therapy significantly accelerated the absorption of pneumonia (*p* = .037), suggesting that the combination of ARB and IFN‐α2b may be beneficial in reducing lung inflammation in patients with mild coronavirus disease 2019 (COVID‐19) but is not effective in accelerating viral clearance (*p* = .057) or reducing the length of hospital stay (*p* = .056).^104^ Chen et al.^105^ included 62 patients with COVID‐19 and divided them into test and control groups according to whether they received ARB during hospitalization. ARB combined with adjuvant therapy could relieve fever in patients with COVID‐19 faster and accelerate the treatment duration to some degree. However, in terms of the length of stay, the differences between ARB and standard therapy were not statistically significant.^105^

Conversely, other studies found no benefit of using ARB in patients with COVID‐19. A study of 86 patients with COVID‐19 revealed that ARB monotherapy has a minor advantage in improving the clinical outcome of patients hospitalized with mild/moderate COVID‐19 over supportive care.^106^ In another study of 81 patients with COVID‐19 in a nonintensive care unit ward comparing patients in the ARB group (*n* = 45) and control group (*n* = 36), which were divided according to the use of ARB, ARB failed to improve the prognosis or accelerate SARS‐CoV‐2 clearance in non‐ICU patients; moreover, the clinical outcomes were significantly better in the control group.^107^ A single‐center randomized controlled trial in Iran included 101 patients with moderate‐to‐severe COVID‐19 and found no significant difference in SARS‐CoV‐2 clearance or prognosis.^108^ ARB is not effective in critically ill patients with COVID‐19. A study retrospectively analyzed 252 patients with COVID‐19 and showed that the rate of clinical improvement was significantly higher in patients treated with ARB than in those who did not receive ARB in terms of moderately and severely ill patients but with no significant difference in critically ill patients.^109^ Another study enrolled 109 patients with severe COVID‐19, with 23 receiving ARB monotherapy, 10 receiving combination therapy with ARB and OSV, and the rest receiving other antiviral drugs; increased in‐hospital mortality was noted in patients with severe COVID‐19 when ARB was administered alone or in combination with OSV.^110^ However, the discrepancy between the efficacies of ARB in vitro versus clinical trials could be due to the various dosages administered to patients.^108^ In vitro studies use high drug doses to obtain inhibition effects; however, in clinical trials, patients cannot be prescribed high doses due to the possibility of side effects.^108^, ^111^ The efficacy of ARB remains controversial; thus, more high‐quality studies are required to establish the efficacy and safety of ARB for COVID‐19.

#### 3.2.3 Anti‐other viruses

ARB has inhibitory effects on many other viruses such as RSV, human adenovirus, CV, parainfluenza virus, rhinovirus, Hantaan virus, chikungunya virus, Lassa virus (LASV), Ebola virus (EBOV), HSV, and hepatitis B and C viruses. However, most studies on the inhibitory effects of ARB on viruses other than influenza and SARS‐CoV‐2 are animal experiments or at the in vitro stage; thus, clinical trial studies should be supported to promote the application of ARB.

RSV belongs to the family *Paramyxoviridae* and is an enveloped negative‐stranded RNA virus, which can cause upper respiratory tract infections and even severe bronchitis and pneumonia. In a mouse model, it was confirmed that the oral ARB 10 and 50 mg kg^−1^ d^−1^ groups had significantly lower titers of RSV than the control group, indicating that ARB has an antiviral effect on RSV in vivo.^83^ Adenovirus is an envelope‐free DNA virus of the family *Adenoviridae* that causes pneumonia and upper respiratory tract infections, with human adenovirus types 3 and 7 being the main pathogens of adenovirus pneumonia. Currently, no clinically recommended therapeutic agents are available. In the HEP‐2 cell assay, ARB has an inhibitory effect only when added after human adenovirus type 7; thus, it is concluded that ARB exerts its antihuman adenovirus type 7 effect in vitro by inhibiting a part of virus biosynthesis.^112^ Furthermore, ARB has shown inhibitory effects on parainfluenza and rhinovirus in vitro cellular assays. ARB was added before, during, and after the infection of HEL cells with rhinovirus type 14 and was found to have an inhibitory effect at all stages. The 50% median effective concentration for parainfluenza virus type 3 was 4.9 μg mL^−1^ and the therapeutic index was 3.5.^83^

ARB has significant inhibitory effects on CVs and HSVs, which cause respiratory symptoms. CV B5, a member of the family *Picornaviridae*, is responsible for several conditions, including respiratory infections, myocarditis, or encephalitis. In a study on the antiviral effects of ARB in mice infected with CV B5, the mice developed interstitial pneumonia and myocarditis; and some mice received oral treatment with ARB for 6 days. At a dose of 50 mg/kg, the drug prolonged survival and reduced the spread of CV in the lungs and heart.^113^ HSV can be divided into HSV‐1 and ‐2 based on the difference in antigenicity. HSV‐1 mainly causes herpetic stomatitis, dermatitis, and encephalitis, whereas HSV‐2 may be associated with cervical cancer. In vitro experiments in human epidermal cells revealed that ARB significantly inhibited HSV‐1 when added before and during viral infection and induced IL‐6 and TNF‐α expression.^81^ Li et al.^63^ observed that ARB significantly reduced skin damage in guinea pigs infected with HSV‐1. Two other studies showed that ABR also inhibited HSV‐2 in vitro; in vivo experiments in mice displayed that either oral administration or use of ointment preparations of ARB controlled the expression of inflammatory cytokines, prolonged the survival time, reduced genital tract injury, and improved the survival rate of HSV‐2‐infected mice.^80^, ^114^

ARB also prevents hemorrhagic fever viruses. Chinese studies have demonstrated the antiviral activity of ARB against Hantaan virus.^82^, ^115^ In vitro, ARB was effective when added before or after viral infection, with IC50 of 0.9 and 1.2 μg mL^−1^, respectively.^82^ In a mouse model experiment of Hantaan virus, ARB was found to have comparable efficacy with ribavirin.^115^ In a study of pseudotyped murine leukemia viruses integrating EBOV or LASV glycoprotein, ARB inhibited the EBOV or LASV GP‐mediated entry process, the average percent inhibition caused by 20 μM ARB was 72.5% in LASV plaque reduction assays, indicating that ARB can inhibit LASV infection by preventing LASV GP‐mediated cell–cell fusion and virus–cell fusion.^116^ Delogu et al.^117^ reported a strong inhibitory effect of ARB on proliferating chikungunya virus in Vero cells and primary human fibroblasts (IC50 < 1. 0 μg·mL^−1^). Di Mola et al.^118^ identified two new ARB derivatives, IIIe and IIIf, with in vitro antiviral activities superior (therapeutic indices = 13.2 and 14.6, respectively) to that of the parent compound ARB (therapeutic index = 4.6). Structural modification of ARB for assessing the conformational relationship of ARB against different physiological activities may be a direction to develop derivatives with better antiviral activity.

As early as 2006, ARB and its derivatives inhibited hepatitis B virus replication in vitro and reduced surface antigen production.^119^ ARB also inhibits hepatitis B virus DNA levels in the HepAD38 and HepG2.2.15 cells in vitro, reduces core dimer levels, and inhibits viral capsid formation in a dose‐dependent manner.^120^ ARB is also effective against the hepatitis C virus of the family *Flaviviridae*,^121^ with the highest viral inhibition efficiency before and during infection.^66^ In vitro experiments showed that ARB inhibited the virus entry and membrane fusion at around 10 μg mL^−1^. ARB obstructed hepatitis C virus entry into cells by affecting lattice‐protein‐mediated endocytosis.^90^, ^122^

ARB also inhibits the foot‐and‐mouth disease virus, Zika virus, and blistering stomatitis virus in in vitro experiments, and the inhibitory effect was more significant when added before or during the infection.^73^, ^123^, ^124^

### 3.3 Safety of ARB

ARB has shown a high safety profile in animal toxicity studies. The 50% lethal doses of ARB in rats and guinea pigs were 3000 and 4000 mg kg^−1^, respectively,^125^ and no biochemical and pathological abnormalities were observed in rats, guinea pigs, rabbits, and dogs after 2–6 months of oral administration of ARB. The oral administration of ARB in rats at doses of 80–320 mg kg^−1^ for 4 weeks showed no significant effects on reproductive function and fetal growth and development of pregnant rats.^126^

ARB is well tolerated and safe to use in humans and no serious drug‐related adverse events have been reported over the years of its use in Russia and China. In a controlled study in Russia, 39 healthy volunteers and 22 patients with acute respiratory disease were given an oral placebo and ARB (dose: 200–800 mg) for 10 days with follow‐up for 10–15 days; no significant difference was observed in the results of various examinations between the two groups.^125^ Another multicenter, randomized, double‐blind, placebo‐controlled phase IV clinical trial conducted in Russia also showed that 42 adverse events occurred in the participants, all were determined to be unrelated to ARB or placebo.^96^ The results from a single‐center, randomized, double‐blind, placebo‐controlled clinical trial in China also showed that the most common adverse reaction of oral ARB 200 mg three times a day was gastrointestinal symptoms, followed by dizziness, which improved after discontinuation of the drug.^127^ Other ARB studies in the treatment of SARS‐CoV‐2 infection^102^, ^104^, ^105^, ^107^, ^128^ have shown no severe adverse events during treatment, with some patients complaining of nausea and stomach pain; yet, no patients discontinued treatment prematurely, suggesting that ARB is a safe and well‐tolerated drug.

### 3.4 Resistance of virus to ARB

ARB has been used for many years in Russia and China for the prevention and treatment of influenza; however, no highly drug‐resistant strains of the virus have been detected. Influenza A/H1N1 and A/H3N2 pandemic strains isolated in Russia in 2008–2009 showed resistance to OSV and amantadine; however, both were sensitive to ARB.^91^, ^129^ In 2011–2012, all tested strains of influenza A and B viruses in the Russian pandemic were susceptible to ARB.^130^ Influenza A and B virus strains isolated in the ARBITR clinical trials from 2012 to 2014 were analyzed via Leneva; ARB was susceptible to all of these strains, including OSV‐resistant strains, and influenza virus strains isolated before and during ARB treatment remained highly susceptible to ARB and NA inhibitors.^131^

However, several influenza strains are resistant to ARB, particularly in the population of influenza B virus strains.^85^ Leneva isolated seven viral mutants from 15 generations of the influenza A/H7N7 strain, all of which showed a single mutation in HA2,^129^ and Nasser et al.^75^ further found that the mutation was restricted to the region 104–120 on the residue HA2, which contained the mutational resistance K117R of ARB. These data suggest that influenza virus resistance to ARB is mainly derived from mutations in HA2 fusion protein and associated with the antiviral activity of ARB with membrane fusion; further studies on ARB resistance to other virus infections are warranted.

## 4 CONCLUSION

The current clinical options for acute viral respiratory infections are limited, highly viral gene mutation has underscored the necessity of novel antiviral drug development. Unlike other antiviral drugs, ARB is a non‐nucleoside broad‐spectrum antiviral drug that not only prevents viral fusion and replication, but also induces the production of interferon, enhances phagocytosis, and other immunomodulatory effects. ARB has been clinically used for the prevention and treatment of influenza A and B, with a good profile of safety and low drug resistance, and it can be used as a pharmacologic enhancer in combination with other drugs since the mechanism of antiviral action of ARB is different from that of other approved antiviral drugs. Based on aforementioned, ARB is a promising drug for clinical application in the prevention and treatment of respiratory viral infections, and the antiviral mechanisms of ARB deserve to be further explored.

However, the research of ARB on many other viruses, such as SARS‐CoV, MERS‐CoV, RSV, CV and Hantaan virus, LASV, etc., mostly not yet in clinical stage, and the conclusions of the current clinical trials on the treatment of COVID‐19 with ARB are still inconsistent. In addition, ARB is currently only produced and used in a few countries, such as China and Russia, and there is a lack of data on the clinical studies on the use of the drug in other countries and populations, so the research on high‐quality clinical trials should be strengthened, especially the global multicenter clinical trial to further confirm its effectiveness and safety and to promote its application.

## AUTHOR CONTRIBUTIONS

**Yue Kang**: Conceptualization; data curation; investigation; resources; writing—original draft. **Yin Shi**: Formal analysis; investigation; resources; visualization; writing—original draft. **Silu Xu**: Conceptualization; methodology; project administration; supervision; validation; writing—review and editing.

## CONFLICT OF INTEREST STATEMENT

The authors declare no conflict of interest.

## Data Availability

Data sharing not applicable to this article as no data sets were generated or analyzed during the current study.
